# Cutting down, quitting and motivation to stop smoking by self‐reported COVID‐19 status: Representative cross‐sectional surveys in England

**DOI:** 10.1111/add.16029

**Published:** 2022-08-26

**Authors:** Sharon Cox, Harry Tattan‐Birch, Sarah E. Jackson, Lynne Dawkins, Jamie Brown, Lion Shahab

**Affiliations:** ^1^ Department of Behavioural Science and Health University College London London UK; ^2^ Spectrum Research Consortium Usher Institute, Old Medical School, University of Edinburgh Edinburgh UK; ^3^ Centre for Addictive Behaviours Research London South Bank University London UK

**Keywords:** COVID‐19, motivation, pandemic, quit attempts, smoking cessation, tobacco cessation

## Abstract

**Aim:**

To examine the association of self‐reported COVID‐19 disease status with cutting down, past‐month and past‐year quit attempts and motivation to stop smoking.

**Design and setting:**

Repeat cross‐sectional survey, representative of the adult population in England.

**Participants:**

Past‐year smokers, *n* = 3338 (aged ≥ 18 years) responding between May 2020 and April 2021.

**Measurements:**

Outcomes were (i) currently cutting down, (ii) having made a quit attempt in the past month, (iii) having made a quit attempt in the past year and (iv) motivation to stop smoking. The explanatory variable was self‐reported COVID‐19 disease status (belief in never versus ever had COVID‐19). Covariates included age, sex, occupational grade, region, children in the household, alcohol use and survey month.

**Findings:**

Of past‐year smokers, 720 (21.6%) reported past‐COVID‐19 infection and 48 (1.4%) reported current COVID‐19 infection. In adjusted analyses, rates of currently cutting down [adjusted odds ratio (aOR) = 1.12, 95% confidence interval (CI) = 0.93–1.34], past‐year quit attempts (aOR = 0.99, 95% CI = 0.82–1.19) and motivation to stop smoking (aOR = 1.04, 95% CI = 0.89–1.23) were comparable in those who did and did not report ever having had COVID‐19. People who reported ever having had COVID‐19 had 39% higher odds than those without of attempting to quit in the past month, but the confidence interval contained the possibility of no difference (aOR = 1.39, 95% CI = 0.94–2.06) and for some the quit attempt may have occurred before they had COVID‐19.

**Conclusion:**

During the first year of the COVID‐19 pandemic in England, rates of reducing smoking and attempting to quit in the past year were similar in smokers who did or did not self‐report ever having had COVID‐19. There was also little difference in motivation to stop smoking between groups. However, causal interpretation is limited by the study design, and there is potential misclassification of the temporal sequence of infection and changes to smoking behaviour.

## INTRODUCTION

Severe acute respiratory syndrome coronavirus 2 (SARS‐CoV‐2 is) an infection that can lead to coronavirus disease (COVID‐19). The SARS‐CoV‐2 pandemic (also known as the COVID‐19 pandemic) has created enormous public health challenges, resulting from the threat of disease and its impact on other health behaviours. Establishing how smoking outcomes have changed since the pandemic is important for assessing its wider impact upon public health goals. In addition, understanding whether having—or believing one has had—COVID‐19 affects people's smoking and quitting behaviour can inform behaviour change theories and provision of cessation support. In this study, we examined associations between self‐reported COVID‐19 status and cutting down, quit attempts and motivation to stop smoking among past‐year smokers in England.

The impact of lockdown restrictions on daily routines (e.g. working from home, reduced social contact) may have helped smokers to avoid their usual triggers and provided an opportune moment to break the habit. Conversely, the ability to smoke without environmental (e.g. work‐place) and other restrictions may have led some people to increase the number of cigarettes they smoke per day. Studies have documented substantial increases in the rates of quit attempts and quit success among smokers since the pandemic began [1]. Some smokers report having cut down the amount they smoke since the pandemic, while others report smoking more [1, 2, 3, 4]. However, the specific experience of being diagnosed with, or believing one has, or COVID‐19 may impact quit motivation in a positive or negative way, independently of the wider effects of the pandemic. Specific to smoking, on one hand, having the disease may trigger an attempt to cut down or stop smoking completely if, for instance, people believe smoking increases their risk of being hospitalized or dying from COVID‐19. On the other hand, already having had the disease and successfully recovering may make people feel there is little reason to change the amount they smoke.

Evidence from the United Kingdom suggests that people who smoke are more concerned about becoming infected with the virus than those who have never smoked, and smokers report being less able to enact personal protective behaviours [5]. However, another study has recently shown that smokers are less likely to perceive smoking as a risk factor for COVID‐19 compared with non‐smokers, reflecting an ‘optimism bias’ of one's own health behaviours [6]. However, perceived probability of contracting the virus appears to be associated with increased motivation to stop smoking [7, 8]. As new studies emerge, the picture on how smoking behaviour has been affected by the pandemic is mixed. To date, little is known about how having had the COVID‐19 disease influences changes in smoking, but fear and experience of illness are a known trigger for quit attempts (e.g. [9, 10]).

The aim of the current study was to characterize changes in smoking behaviour and motivation to stop among a representative sample of past‐year smokers in England surveyed between May 2020 and April 2021. COVID‐19 disease status was measured by self‐report belief in ever having the disease (i.e. not a positive test).

Specifically, we sought to address the following research questions:

1. Among current smokers, what is the association between self‐reported COVID‐19 status (ever, including current and past disease versus never) and currently cutting down or having made a quit attempt in the past month, after adjusting for covariates?
2. Among past‐year smokers, what is the association between self‐reported COVID‐19 status (past versus never) and past‐year quit attempts, after adjusting for covariates?
3. Among current smokers, what is the association between self‐reported COVID‐19 status (ever versus never) and motivation to stop smoking, after adjusting for covariates?

## METHODS

### Study design, setting and participants

Data were collected between May 2020 and April 2021 from the Smoking Toolkit Study (STS), a monthly repeated cross‐sectional survey representative of adults in England [11]. This report follows Strengthening The Reporting of Observational Studies in Epidemiology (STROBE) guidelines for cross‐sectional studies [12].

The STS uses a hybrid of random location and quota sampling to select a new sample of approximately 1700 adults (aged ≥ 18 years) each month (see [10] for details). Prior to the pandemic, data were collected face‐to‐face and switched to telephone during this time (and the whole time of this study). The same sampling approach was used for the face‐to‐face method and for which we have shown yields similar estimates of key parameters to other national surveys in England and sales data. We have subsequently reported diagnostic analyses in other papers, which suggest that there is continuity in estimates across the approaches (e.g. [1]). Survey weights are calculated using raking to adjust the sample to match the population of England based on key demographic variables. This demographic profile is determined using a combination of data from the 2011 UK Census, Office for National Statistics mid‐year estimates and the National Readership Survey.

Respondents who answered the question relating to COVID‐19 symptoms and affirmed past‐year tobacco smoking were included in the study. The COVID‐19 symptom questions were introduced in the STS in March 2020; the list of symptoms was based on the UK Government's advice at that time. As more was known about the disease, other common symptoms were associated with the disease (e.g. anosmia) but these symptoms were not added to the STS. May 2020 was selected as the start date because this is when widespread access and testing became easily available to people in England with COVID‐19 symptoms, meaning that the accuracy of whether people were infected was likely to be greater than earlier in the pandemic, when a greater proportion of cases were not confirmed by testing. April 2021 was the end‐point for our COVID‐19 data collection. During this period, England experienced two national lockdowns resulting in continued social restriction. This time predated the widespread COVID‐19 vaccination programme across all age groups. Details of the UK COVID‐19 prevalence rate during the time‐period of this study can be found elsewhere [13]. Ethical approval for the STS was granted by the UCL Ethics Committee (ID 0498/001).

## VARIABLES

### Explanatory variables: smoking status

Smoking status was determined by asking: ‘Which of the following best applies to you?’: (1) I smoke cigarettes (including hand‐rolled) every day; (2) I smoke cigarettes (including hand‐rolled), but not every day; (3) I do not smoke cigarettes at all, but I do smoke tobacco of some kind (e.g. pipe, cigar or shisha); (4) I have stopped smoking completely in the last year; (5) I stopped smoking completely more than a year ago; and (6) I have never been a smoker (i.e. smoked for a year or more). Current smokers were defined as those who responded 1–3, while past‐year smokers were those who responded 1–4. All others were considered non‐smokers.

### Self‐reported COVID‐19

Belief to have ever had COVID‐19 disease was determined by participants being told that ‘the key symptoms for coronavirus are high temperature/fever or a new, continuous cough’. They were then asked which of the following statements best applied to them: (1) ‘I definitely have coronavirus’, (2) ‘I think I have coronavirus’, (3) ‘I definitely had coronavirus’, (4) ‘I think I had coronavirus’, (5) ‘I do not have or think I have had coronavirus’, (6) ‘do not know’ and (7) ‘prefer not to say’. These responses were recoded as follows: currently have/currently think I have COVID‐19 as ‘current’, definitely/think had COVID‐19 as ‘past’ or a combination of these two as ‘ever COVID‐19’ and do not think I have/had as ‘never’; 6 and 7 were excluded from the analysis.

### Covariates

Socio‐demographic covariates included were: sex, age, region and occupational social grade dichotomized into ABC1 (managerial, professional and clerical workers) and C2DE (manual and casual workers, state pensioners and unemployed), as derived from the National Readership Survey [11]. In order to account for confounding factors that are associated with smoking and self‐reported COVID‐19, we also adjusted for presence of children in the house (yes/no) [14], AUDIT‐C [12], a three‐item brief measure of alcohol consumption (analysed as a continuous variable [1]) and survey month in the analysis. Use of nicotine replacement products and e‐cigarettes were not included as covariates as these were considered potential mediators rather than confounders.

### Outcome variable

Current smokers were asked: ‘Are you currently trying to cut down on how much you smoke?’, (yes/no) and ‘Have you made a quit attempt in the past month?’ (yes/no). Their motivation to stop smoking was assessed using the motivation to stop scale [15]; a single‐item continuous measure which asks: ‘Which of the following best describes you?’. Responses include: ‘I *really* want to stop smoking and intend to in the next month’, ‘I *really* want to stop smoking and intend to in the next 3 months’, ‘I want to stop smoking and hope to soon’, ‘I *really* want to stop smoking but I do not know when I will’, ‘I want to stop smoking but have not thought about when’, ‘I think I should stop smoking but do not really want to’ and ‘I do not want to stop smoking’.

Past‐year smokers were asked: ‘Have you made a serious quit attempt in the past 12 months?’ (yes/no). We were not able to ascertain from this measure if past‐year smokers quit attempts preceded the pandemic.

#### Statistical method

This study protocol was preregistered on the Open Science Framework (https://osf.io/h5qf8). Several changes to the registered protocol were made. Housing status was not included, as this information was not collected during the study period. Research questions 1 and 3 were meant to be assessed separately in those with past and current COVID‐19 disease, but owing to low participant numbers in the latter we changed this to ‘never’ versus ‘ever having had COVID’ (i.e. combining past and current COVID‐19 disease self‐reports). All three COVID‐19 disease statuses by outcome measure and smoking status are still provided descriptively. After internal review, we ran a sensitivity analysis where we included strength of urges to smoke (a known marker of tobacco dependence and relapse [16]) as an additional covariate (https://osf.io/h5qf8).

Data were analysed on complete cases using R version 4.1.1. The level of missing data was not problematic; 0.5% participants had missing smoking status data, 2.1% did not respond to the question about COVID‐19 and missing data was < 5% for all other variables. We present weighted descriptive statistics [% (*n*) or means ± standard deviations (SDs)] to report the participant characteristics by COVID‐19 disease status.

We conducted logistic regressions to estimate associations between COVID‐19 disease status and (i) currently cutting down, (ii) making a past‐year or past‐month quit attempt and (iii) motivation to stop smoking. As motivation to stop is an ordinal variable, this was analysed using ordered logistic regression. The main associations are presented with and without adjustment for covariates. We adjusted for survey month to account for changes in quitting motivation and behaviour and in the incidence of COVID‐19 over time, using restricted cubic splines with three knots placed at quantiles to allow for non‐linear trends. Bayes factors (BF) for non‐significant results are available on‐line (https://osf.io/h5qf8).

## RESULTS

### Participant and descriptive data

A total of 19 544 participants responded during the study period, 3338 of whom (17.1%) were past‐year smokers and 2923 of whom (87.6%) were current smokers. Of past‐year smokers, 2490 (74.6%) reported never having contracted COVID‐19, 720 (21.6%) reported past‐COVID‐19 disease, 48 (1.4%) reported a current COVID‐19 disease and 70 (2.1%) reported not knowing or preferring not to say (excluded from subsequent analyses). Weighted sample characteristics for past‐year smokers are presented in Table **1**, stratified by COVID‐19 disease status. Adjusted analyses included data from the 3019 past‐year smokers and 2651 current smokers who responded to all relevant questions.

**TABLE 1:** Characteristics of analysed sample of past‐year smokers by self‐reported COVID‐19 infection status.

	Never COVID *n* = 2689	Past‐COVID *n* = 825	Current COVID *n* = 50	Total *N* = 3564
Age (years)				
16–24	417 (15.5%)	181 (21.9%)	7 (14.3%)	605 (17.0%)
25–34	674 (25.1%)	243 (29.5%)	21 (41.4%)	938 (26.3%)
35–44	506 (18.8%)	152 (18.4%)	7 (14.9%)	665 (18.7%)
45–54	369 (13.7%)	131 (15.9%)	9 (17.0%)	509 (14.3%)
55–64	329 (12.2%)	76 (9.2%)	4 (8.1%)	409 (11.5%)
≥ 65	394 (14.7%)	42 (5.1%)	2 (4.3%)	438 (12.3%)
Female				
Female	1246 (46.3%)	370 (44.9%)	21 (41.0%)	1637 (45.9%)
Social grade				
C2DE	1582 (60.7%)	469 (59.0%)	19 (38.7%)	2070 (60.0%)
Children in household				
No	1899 (70.6%)	548 (66.5%)	31 (62.2%)	2478 (69.5%)
Region				
North	777 (28.9%)	216 (26.1%)	16 (32.4%)	1009 (28.3%)
Central	828 (30.8%)	270 (32.8%)	8 (16.1%)	1107(31.1%)
London	382 (14.2%)	140 (17.0%)	12 (22.9%)	533 (15.0%)
South	702 (26.1%)	199 (24.1%)	14 (28.6%)	916 (25.7%)
Alcohol use (AUDIT‐C)				
Mean (SD)	3.8 (SD = 3.4)	4.5 (SD = 3.4)	3.5 (SD = 3.2)	3.9 (SD = 3.4)
Survey year				
2020	1786 (66.4%)	562 (68.2%)	21 (41.2%)	2369 (66.5%)
2021	903 (33.6%)	263 (31.8%)	30 (58.8%)	1196 (33.5%)
Current smoker				
Yes	2365 (88.0%)	724 (87.8%)	47 (94.0%)	3136 (88.0%)
Currently cutting down^a^				
Yes	1185 (50.7%)	369 (51.2%)	27 (59.8%)	1581 (51.0%)
Past‐month quit attempt^a^				
Yes	116 (4.9%)	42 (5.7%)	5 (9.9%)	162 (5.2%)
Past‐year quit attempt				
Yes	974 (36.2%)	297 (36.0%)	20 (39.2%)	1291 (36.2%)
Motivation to stop (MTSS)^a^				
1 (Do not want to stop)	556 (24.2%)	137 (19.5%)	9 (21.1%)	702 (23.1%)
2	512 (22.3%)	180 (25.7%)	10 (22.5%)	702 (23.0%)
3	297 (12.9%)	100 (14.2%)	6 (14.5%)	403 (13.3%)
4	226 (9.8%)	69 (9.9%)	6 (12.9%)	301 (9.9%)
5	375 (16.3%)	100 (14.3%)	7 (14.8%)	481 (15.8%)
6	205 (8.9%)	75 (10.7%)	2 (5.2%)	282 (9.3%)
7 (Intend to stop in < 1 month)	128 (5.6%)	41 (5.8%)	4 (9.1%)	173 (5.7%)

Data are presented as *n* (%), for categorical variables. Continuous variables (e.g. AUDIT‐C) are presented as mean [standard deviation (SD)].

^a^ Current smokers only. AUDIT‐C = Alcohol use disorders identification test consumption. MTSS = motivation to stop smoking scale. Data are weighted.

## MAIN RESULTS

Half of current smokers reported that they were cutting down, regardless of whether (51.4%) or not (49.6%) they self‐reported ever having had COVID‐19 [adjusted odds ratio (aOR) = 1.12, 95% confidence interval (CI) = 0.93–1.34 for cutting down]. People who reported ever having had COVID‐19 had 39% higher odds than those without COVID‐19 of attempting to quit in the past month, but the CI contained the possibility of no difference (aOR = 1.39, 95% CI = 0.94–2.06; Table 2).

**TABLE 2:** Association of self‐reported COVID‐19 with cutting down and attempts and motivation to stop smoking.

	*n* (%)	OR	95% CI	aOR	95% CI
Cutting down^a^					
Never COVID	1068 (49.6%)	1	–	1	–
Ever COVID^b^	349 (51.4%)	1.07	0.90–1.28	1.12	0.93–1.34
Past‐month quit attempt^a^					
Never COVID	103 (4.7%)	1	–	1	–
Ever COVID^b^	42 (6.1%)	1.32	0.91–1.91	1.39	0.94–2.06
Past‐year quit attempt					
Never COVID	896 (35.3%)	1	–	1	–
Past‐COVID	265 (36.3%)	1.05	0.88–1.24	0.99	0.82–1.19
Motivation to stop (MTSS)^a^					
Never COVID	3.16 (SD = 1.90)	1	–	1	–
Ever COVID^b^	3.27 (SD = 1.89)	1.13	0.97–1.32	1.04	0.89–1.23

CI = confidence interval; aOR = adjusted odds ratio; MTSS = motivation to stop smoking scale. Adjusted for survey month, sex, age, social grade, geographic region, children in the household and AUDIT‐C.

^a^ Current smokers only.

^b^ Ever COVID‐19 includes both current and past‐COVID‐19 infection. *n* (%) and mean [standard deviation (SD)] were calculated using survey weights, but OR and aOR were not.

Just over a third of past‐year smokers reported making a quit attempt in the last year, regardless of whether (36.3%) or not (35.3%) self‐reported having previously had COVID‐19. Odds of making a quit attempt in the last year were similar in both these groups after adjustment for confounders (aOR = 0.99, 95% CI = 0.82–1.19; Table **2**).

There was little difference in motivation to stop smoking among current smokers who reported ever having had COVID‐19 (mean = 3.27, SD = 1.90) and those without any past or current disease (mean = 3.16, SD = 1.89). The CI from unadjusted ordinal regression excluded large differences (OR = 1.13, 95% CI = 0.97–1.32), and the odds ratio weakened towards the null after adjustment for measured confounders (aOR = 1.04, 95% CI = 0.89–1.23: Table 2).

## DISCUSSION

Our data show that, among smokers in England, rates of cutting down, attempts to quit and motivation to stop smoking were similar in those who did and did not report ever having had COVID‐19. One interpretation would be that infection has little direct effect on people's likelihood of attempting to quit smoking or cut down. However, several alternative explanations must be considered. First, the impact of disease could vary throughout individuals: being infected, or at least believing one has been infected, may trigger quit attempts in some people, while others who recover may believe they are now immune, making them less concerned about the effect of smoking on their health than those who have never been infected. These competing effects could cancel each other out, leading us to observe little association overall. Secondly, because our data were cross‐sectional, some past‐year quit attempts will undoubtedly have occurred prior to disease. This would have been less of an issue although not completely resolved for past‐month quit attempts, as these are less likely to have occurred before people had COVID‐19 than past‐year quit attempts. Such temporal misclassification could have biased results towards the null, as could residual confounding [17]. Finally, while most confidence intervals excluded large associations with COVID‐19 status—with an upper aOR limit less than 1.34 for all outcomes except past‐month quit attempts—all include the possibility of small differences. Further studies are needed to estimate these associations with greater precision.

There are some limitations to the current study. The data were cross‐sectional, which meant that we could not determine whether participants attempted to quit smoking before or after having the disease. Survey measures may miss some of the nuances which have led to people to change their smoking (e.g. disease severity, long COVID, unemployment, home learning), thus qualitative data could supplement our understanding of the unique challenges of quitting smoking within this context. We were not able to account for disease severity nor the effects of long COVID, which may well lead to differences in both quit attempts and sustained attempts; future research stratified on severity of symptoms and accounting for long‐term symptoms could elucidate these types of important differences. While we included covariates of social economic positioning and family status, there may be residual confounding. For example, we know that important contextual and intersecting factors, such as employment type [18, 19] and financial stability [20], accommodation circumstances (e.g. secure accommodation, suitability of accommodation for lockdown [21, 22] and ethnicity [18, 23] have been strongly associated with elevated risk of contracting COVID‐19, and health comorbidities with disease severity [24], all of which could influence beliefs of having contracted COVID‐19 and, potentially, smoking outcomes. Lastly, our measure of COVID‐19 was developed in the early phase of the pandemic, before more common symptoms (e.g. anosmia) were widely understood be associated with the having been infected and prior to widespread testing being available.

The results presented have little implication for theory, potentially highlighting the complexity of smoking cessation in the context of wider health behaviours which need to be explored with greater empirical precision. However, there are some policy considerations. There is a lack of evidence that having COVID‐19 acts as a widespread teachable moment for smoking cessation, at least in the long term, so there is a need for continued investment in tobacco control and cessation support. This is especially the case for priority groups with a high smoking prevalence, for which more targeted support is already urgently needed; whether the pandemic has slowed progress in supporting these groups to quit remains unknown. Practice may also need to focus upon those who have compromised respiratory symptoms because of COVID‐19 and who also smoke; this is a new type of vulnerability and challenge for practitioners, although there are some signs that stop‐smoking services are doing this but on an individual, rather than systems, level [25].

## CONCLUSIONS

During the first year of the COVID‐19 pandemic in England, rates of cutting down and attempting to quit were similar in smokers who did versus did not self‐report that they had ever been infected with COVID‐19. There was also little difference in motivation to stop between groups. However, these results are caveated with the limitation that we cannot be sure whether participants changed their behaviour before or after having the disease, so the direction of causality is unclear.

## DECLARATION OF INTERESTS

S.C., H.T.B., S.E.J. and L.D. have no competing interests. J.B. has received unrestricted research funding to study smoking cessation from manufacturers of smoking cessation medications (Pfizer and Johnson & Johnson). L.S. is a HEFCE‐funded member of staff at University College London. He has received honoraria for talks, an unrestricted research grant and travel expenses to attend meetings and workshops from Pfizer and an honorarium to sit on advisory panel from Johnson & Johnson, both pharmaceutical companies that make smoking cessation products.

## AUTHOR CONTRIBUTIONS

**Sharon Cox:** Conceptualization; data curation; formal analysis; methodology; project administration. **Harry Tattan‐Birch:** Data curation; formal analysis; investigation; methodology; project administration. **Sarah Jackson:** Conceptualization; formal analysis; investigation. **Lynne Dawkins:** Conceptualization; investigation; methodology. **Jamie Brown:** Conceptualization; formal analysis; funding acquisition; methodology. **Lion Shahab:** Conceptualization; formal analysis; funding acquisition; investigation; methodology; project administration; supervision.

## References

[1] Jackson SE , Garnett C , Shahab L , Oldham M , Brown J . Association of the COVID‐19 lockdown with smoking, drinking and attempts to quit in England: an analysis of 2019–20 data. Addiction. 2021;16:1233–44. 10.1111/add.15295 PMC8436745 33089562

[2] Tattan‐Birch H , Perski O , Jackson S , Shahab L , West R , Brown J . COVID‐19, smoking, vaping and quitting: a representative population survey in England. Addiction. 2021;116:1186–95. 32918300 10.1111/add.15251 PMC8436761

[3] Siddiqi K , Siddiqui F , Khan A , Ansaari S , Kanaan M , Khokhar M , et al. The impact of COVID‐19 on smoking patterns in Pakistan: findings from a longitudinal survey of smokers. Nicotine Tob Res. 2021;23:765–9. 33029618 10.1093/ntr/ntaa207 PMC7665599

[4] Kale D , Perski O , Herbec A , Beard E , Shahab L . Changes in cigarette smoking and vaping in response to the COVID‐19 pandemic in the UK: findings from baseline and 12‐month follow up of HEBECO study. Int J Environment Res Public Health. 2022;19:630. 10.3390/ijerph19020630 PMC8775930 35055451

[5] Jackson SE , Brown J , Shahab L , Steptoe A , Fancourt D . COVID‐19, smoking and inequalities: a study of 53 002 adults in the UK. Tob Control. 2020;e111–21. 10.1136/tobaccocontrol-2020-055933 32826387

[6] Herbec A , Brown J , Jackson SE , Kale D , Zatoński M , Garnett C , et al. Perceived risk factors for severe Covid‐19 symptoms and their association with health behaviours: findings from the HEBECO study. Acta Psychol. 2022;222:103458. 10.1016/j.actpsy.2021.103458 PMC8639445 34933210

[7] Brown CRH . The relationship between COVID‐19‐specific health risk beliefs and the motivation to quit smoking: a UK‐based survey. Drug Alcohol Depend. 2021;227:108981. 34488076 10.1016/j.drugalcdep.2021.108981 PMC8397491

[8] Gravely S , Craig LV , Cummings KM , Ouimet J , Loewen R , Martin N , et al. Smokers’ cognitive and behavioural reactions during the early phase of the COVID‐19 pandemic: findings from the 2020 ITC four country smoking and vaping survey. PLOS ONE. 2021;16:e0252427. 34086706 10.1371/journal.pone.0252427 PMC8177641

[9] Vangeli E , Stapleton J , Smit ES , Borland R , West R . Predictors of attempts to stop smoking and their success in adult general population samples: a systematic review: predictors of quit attempts and success. Addiction. 2011;106:2110–21. 21752135 10.1111/j.1360-0443.2011.03565.x

[10] Im PK , McNeill A , Thompson ME , Fong GT , Xu S , Quah ACK , et al. Individual and interpersonal triggers to quit smoking in China: a cross‐sectional analysis. Tob Control. 2015;24:iv40–7. 25888422 10.1136/tobaccocontrol-2014-052198 PMC4644698

[11] Fidler JA , Shahab L , West O , Jarvis MJ , McEwen A , Stapleton JA , et al. ‘The smoking toolkit study’: a national study of smoking and smoking cessation in England. BMC Public Health. 2011;11:479. 21682915 10.1186/1471-2458-11-479 PMC3145589

[12] von Elm E , Altman DG , Egger M , Pocock SJ , Gøtzsche PC , Vandenbroucke JP , et al. The strengthening the reporting of observational studies in epidemiology (STROBE) statement: guidelines for reporting observational studies. Ann Intern Med. 2007;147:573–7. 17938396 10.7326/0003-4819-147-8-200710160-00010

[13] Coronavirus (COVID‐19) latest insights: Infections [internet]. Office for National Statistics; 2022 [cited 2022 Apr 20]. Available at: https://www.ons.gov.uk/peoplepopulationandcommunity/healthandsocialcare/conditionsanddiseases/articles/coronaviruscovid19latestinsights/infections Accessed 13 Jul 2022.

[14] Kock L , Brown J , Shahab L , Tattan‐Birch H , Moore G , Cox S . Inequalities in smoking and quitting‐related outcomes among adults with and without children in the household 2013–2019: a population survey in England. Nicotine Tob Res. 2022;24:690–8. 34634112 10.1093/ntr/ntab211 PMC8962729

[15] Kotz D , Brown J , West R . Predictive validity of the motivation to stop scale (MTSS): a single‐item measure of motivation to stop smoking. Drug Alcohol Depend. 2013;128:15–9. 22943961 10.1016/j.drugalcdep.2012.07.012

[16] Doherty K , Kinnunen T , Militello FS , Garvey AJ . Urges to smoke during the first month of abstinence: Relationship to relapse and predictors. Psychopharmacology. 1995;119:171–8. 7659764 10.1007/BF02246158

[17] Rothman K , Greenland S , Lash T . Modern Epidemiology 3rd ed. 3 Philadelphia, PA: Wolters Kluwer Health/Lippincott Williams & Wilkins; 2008.

[18] Selden TM , Berdahl TA . COVID‐19 and racial/ethnic disparities in health risk, employment, and household composition: study examines potential explanations for racial‐ethnic disparities in COVID‐19 hospitalizations and mortality. Health Aff. 2020;39:1624–32. 10.1377/hlthaff.2020.00897 32663045

[19] Houlihan CF , Vora N , Byrne T , Lewer D , Kelly G , Heaney J , et al. Lancet. 2020;396:e6–7.32653078

[20] Marmot M . Health equity in England: the Marmot review 10 years on. BMJ. 2020;368:m693. 32094110 10.1136/bmj.m693

[21] Lewer D , Braithwaite I , Bullock M , Eyre MT , White PJ , Aldridge RW , et al. COVID‐19 among people experiencing homelessness in England: a modelling study. Lancet Respir Med. 2020;8:1181–91. 32979308 10.1016/S2213-2600(20)30396-9 PMC7511167

[22] Baggett TP , Keyes H , Sporn N , Gaeta JM . Prevalence of SARS‐CoV‐2 infection in residents of a large homeless shelter in Boston. JAMA. 2020;323:2191–2. 32338732 10.1001/jama.2020.6887 PMC7186911

[23] Aldridge RW , Lewer D , Katikireddi SV , Mathur R , Pathak N , Burns R , et al. Black, Asian and minority ethnic groups in England are at increased risk of death from COVID‐19: indirect standardisation of NHS mortality data. Wellcome Open Res. 2020;5:88. 32613083 10.12688/wellcomeopenres.15922.1 PMC7317462

[24] Wang B , Li R , Lu Z , Huang Y . Does comorbidity increase the risk of patients with COVID‐19: evidence from meta‐analysis. Aging. 2020;12:6049–57. 32267833 10.18632/aging.103000 PMC7185114

[25] Cox S , Ward E , Ross L , Notley C . How a sample of English stop smoking services and vape shops adapted during the early COVID‐19 pandemic: a mixed‐methods cross‐sectional survey. Harm Reduct J. 2021;18:95. 34465346 10.1186/s12954-021-00541-0 PMC8406006

